# Salad Leaf Juices Enhance Salmonella Growth, Colonization of Fresh Produce, and Virulence

**DOI:** 10.1128/AEM.02416-16

**Published:** 2016-12-15

**Authors:** Giannis Koukkidis, Richard Haigh, Natalie Allcock, Suzanne Jordan, Primrose Freestone

**Affiliations:** a Department of Infection, Immunity and Inflammation, University of Leicester, Leicester, United Kingdom; b Department of Genetics, University of Leicester, Leicester, United Kingdom; c Core Biotechnology Services, University of Leicester, Leicester, United Kingdom; d Campden BRI, Chipping Campden, Gloucestershire, United Kingdom; University of Helsinki

**Keywords:** Salmonella, fresh produce, biofilm, motility, salad leaf colonization

## Abstract

We show in this report that traces of juices released from salad leaves as they become damaged can significantly enhance colonization of salad leaves by Salmonella enterica. Salad juices in water increased Salmonella growth by 110% over the level seen with the unsupplemented control and in host-like serum-based media by more than 2,400-fold over control levels. In serum-based media, salad juices induced growth of Salmonella via provision of Fe from transferrin, and siderophore production was found to be integral to the growth induction process. Other aspects relevant to salad leaf colonization and retention were enhanced, such as motility and biofilm formation, which were increased over control levels by >220% and 250%, respectively; direct attachment to salad leaves increased by >350% when a salad leaf juice was present. In terms of growth and biofilm formation, the endogenous salad leaf microbiota was largely unresponsive to leaf juice, suggesting that Salmonella gains a marked growth advantage from fluids released by salad leaf damage. Salad leaf juices also enhanced pathogen attachment to the salad bag plastic. Over 5 days of refrigeration (a typical storage time for bagged salad leaves), even traces of juice within the salad bag fluids increased Salmonella growth in water by up to 280-fold over control cultures, as well as enhancing salad bag colonization, which could be an unappreciated factor in retention of pathogens in fresh produce. Collectively, the study data show that exposure to salad leaf juice may contribute to the persistence of Salmonella on salad leaves and strongly emphasize the importance of ensuring the microbiological safety of fresh produce.

**IMPORTANCE** Salad leaves are an important part of a healthy diet but have been associated in recent years with a growing risk of food poisoning from bacterial pathogens such as Salmonella enterica. Although this is considered a significant public health problem, very little is known about the behavior of Salmonella in the actual salad bag. We show that juices released from the cut ends of the salad leaves enabled the Salmonella cells to grow in water, even when it was refrigerated. Salad juice exposure also helped the Salmonella cells to attach to the salad leaves so strongly that washing could not remove them. Collectively, the results presented in this report show that exposure to even traces of salad leaf juice may contribute to the persistence of Salmonella on salad leaves as well as priming it for establishing an infection in the consumer.

## INTRODUCTION

Fresh produce such as green salad leaves is recognized as an important part of a healthy diet, and its consumption in the European Union and United States has increased considerably in recent years, making such produce an important economic output (1, 2). Salad leaves (lettuces and spinach), because of their high water content, are highly perishable and subject to rapid spoilage caused by endogenous and exogenous microbes and thus require rapid processing and specialist packing (3). Salad leaves are also subject to colonization by enteric pathogens, most frequently Salmonella enterica, Escherichia coli, and Listeria monocytogenes (3–8). Epidemiological profiling now ranks salads as the second most common source of outbreaks of foodborne illness (3, 9, 10). Based on experimental investigations, part of the explanation may be that normally animal-associated pathogens can, via their presence in contaminated soils, directly internalize salad leaves during seed germination by entering the plant vasculature, possibly via the root hairs (11, 12). Salmonella shows chemotaxis toward root exudates and was found to colonize the interior of tomato plants which were grown hydroponically (13, 14). Salmonella is also capable of replicating within leafy green plant tissues as well as on the surface of plant leaves (15, 16). The path of salad leaf production from farm to fork has multiple opportunities for microbial pathogen contact. Harvesting involves cutting of leaves off stalks, trimming, washing, packaging, and transport (3). Contamination of fresh salad produce is thought to occur via routes such as animal or insect contacts, soil, contaminated irrigation and wash waters, and nonhygienic equipment and human handling (3, 17). Practices designed to reduce pathogen growth in fresh salad produce include refrigeration after harvesting and rapid processing and packaging in a modified atmosphere containing reduced oxygen levels (3, 17). Despite all these efforts, salad-associated infections still occur.

In terms of the health burden of fresh salad produce infections, in 2014 in the United States, bean sprouts contaminated with Salmonella infected over 100 people, a quarter of whom were hospitalized (18). Bagged ready-to-eat rocket leaves caused a Salmonella enterica serovar Thompson salmonellosis outbreak in the UK and Scandinavia in 2008 (19), while fresh basil was a source of infection by Salmonella serovar Anatum in Denmark in 2009 (20). In February 2016, more than 50 people in Victoria, Australia, developed salmonellosis after eating bagged salad leaves (21). Due to the increasingly frequent recurrence of fresh produce outbreaks, the European Food Safety Authority (EFSA) Panel on Biological Hazards (17) has classified leafy green salads as among the top sources of foodborne infections within the European Union, with Salmonella being implicated in around 30% of outbreak cases (17).

Foods such as salad leaves pose such a serious infection risk because they are usually minimally processed and consumed raw (3, 5, 6, 8, 9). There is, not surprisingly, much interest in finding ways to prevent pathogen contamination, and considerable research effort has been expended in attempts to improve the microbial safety of salad leaf culture as well as to optimize protocols for processing and packaging. As early as 1998, the US FDA issued guidelines designed to minimize foodborne infection from fresh produce, and the guidelines were developed further in a 2008 WHO directive (3, 22, 23). Decontamination of salad leaves using topical sanitizing agents such organic acids, hydrogen peroxide, or chlorine or using irradiation have been used to control pathogen levels, though such treatments can affect leaf texture and flavor (3). Also, Salmonella internalization into fresh produce can markedly reduce the efficacy of topical sanitizing agents (3). Several research initiatives have looked at the salad leaf microbiome as a means of improving food safety, as salad leaves are colonized by diverse microbiotas, particularly those that are field grown (24). Use of lactic acid bacteria isolated from fresh produce inhibited the growth of S. enterica serovar Typhimurium on fresh-cut lettuce by up to 2 logs/g leaf (3). Other investigations have used bacteriophages in combination with antagonistic bacteria, and Ye et al. (25) showed that combining a Salmonella bacteriophage with an antagonistic Enterobacter isolate from fresh produce reduced the numbers of Salmonella in a broth culture by up to 6 log orders. However, there are as yet no treatments which can completely remove foodborne pathogens from fresh salad produce.

While the reports which explore methods to improve the microbiological safety of salad leaves are many (4–6, 8), fewer studies exist which consider the behavior of Salmonella when they have gained entry into the salad leaf bag. It has been shown that growth of foodborne pathogens on salad leaves is enhanced once the protective epidermal surface is damaged, especially if the salad leaves experience storage at nonrefrigeration temperatures (12). It is also known that salad leaves can become damaged during processing (3) and that they also are often cut or chopped to meet consumer requirements, which, as well as releasing and spreading leaf fluids, could also promote dissemination of any pathogens present. Since it is likely that bacteria in bagged salads would come into contact with leached salad leaf fluids, it is important for food safety reasons to understand whether this changes the behavior of pathogens such as Salmonella, and so this is the focus of the current study.

## RESULTS AND DISCUSSION

### Salad juices enhance Salmonella growth at refrigeration temperatures.

**FIG 1:**
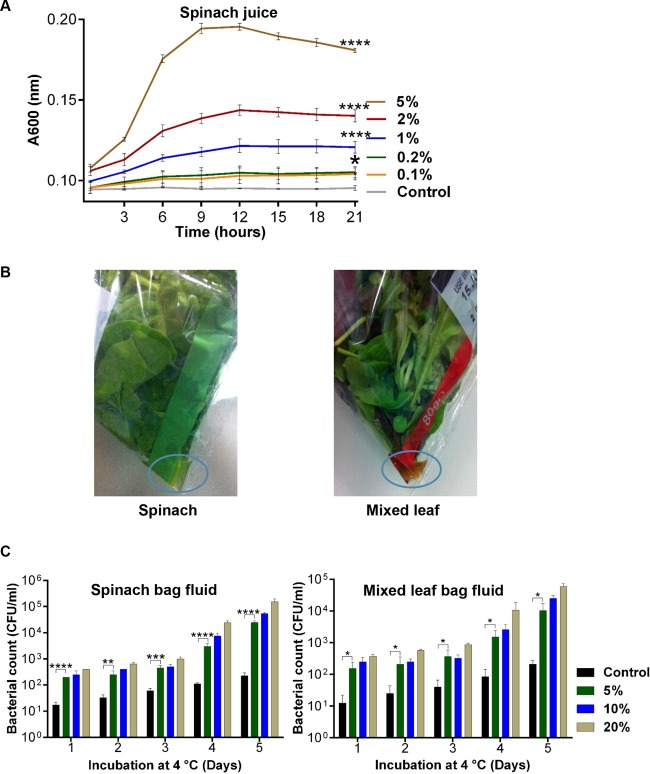
Salad extracts induce Salmonella growth in water. (A) *In vitro* time course of growth at 37°C of Salmonella in water with increasing levels of spinach juice; values shown are means from triplicate time points; *n* = 3. (B) Images showing the fluid that accumulates in bagged salads, spinach, and mixed-leaf varieties. (C) Histograms showing the *in vitro* growth at 4°C of 10^1^ CFU/ml Salmonella inoculated into sterile water supplemented with 5% to 20% (vol/vol) doses of fluids from a spinach and mixed-leaf salad bag; values shown are means from triplicate time points; *n* = 3. *, *P* ≤ 0.05; **, *P* ≤ 0.01; ***, *P* ≤ 0.001; ****, *P* ≤ 0.0001.

Physical damage to fresh produce and cutting of produce have been shown to promote the growth of Salmonella, especially at nonrefrigeration temperatures (3, 4, 6). Compositional analyses of the nutrients within salad leaves such as lettuce and spinach show typical water content of 93 to 96% and total protein and sugar levels of 1.2 to 2.6% and 1.4 to 2.6%, respectively (26); such nutrient levels, while low, are growth supportive. We used a water-only medium to examine salad leaf growth effects because salad leaves are routinely covered with a water film to maintain texture (3). Figure 1A shows a typical result for spinach juice and that growth in water at 37°C was supported in a dose-dependent manner. Similar results were also seen with the other leaf juices (data not shown), all of which produced similar growth enhancement at 2%, which we then adopted as our standard salad juice addition concentration. A recent study by Posada-Izquierdo et al. (27) also examined the growth kinetics of Salmonella in leafy salad extracts (chard, iceberg lettuce, parsley, and spinach). Those authors used much higher levels (33.3% [vol/vol]) of leaf juice in their growth analyses and found growth rates lower than those that we report here. In marked contrast, a report by Segura et al. showed that leaf extracts such as spinach were markedly inhibitory to the growth of both Gram-negative and Gram-positive bacteria, due to the presence of defensin-like antimicrobial peptides (28).

We were also interested in determining what effect the traces of juices released from salad leaves might have on behavior of the pathogen at refrigeration temperatures, as this is the typical environment for storage of a bagged salad. We had noticed that many of the salad bags purchased had fluid within them whose color was similar to but lighter than the color of the leaf juices that we extracted (examples in a spinach bag and a mixed-leaf bag are shown in Fig. 1B), and so we investigated whether the juices were also stimulatory to Salmonella growth at refrigeration temperatures. The results of incubations with spinach bag fluid and mixed-salad bag fluid are shown in Fig. 1C. Over the course of the 5 days at 4°C, the unsupplemented control cultures grew only around 1 log more. However, even though it has been shown that refrigeration temperatures restrict pathogen growth on fresh produce (12) and that the temperature preference of Salmonella is the same as that of its mammalian host, i.e., 37°C, in the presence of spinach or mixed-leaf bag fluid, growth at 4°C was continuous for all of the fluid doses. By the end of 5 days, the levels of Salmonella cultures supplemented with 20% (vol/vol) bag fluid had increased by nearly 3 logs over the controls (*P* ≤ 0.0001). These analyses suggest that Salmonella bacteria contaminating a bagged salad would be able to use the leaf nutrients leached into the bag water film to promote their own proliferation and retention even within a refrigerated environment.

### Salmonella motility and biofilm formation are enhanced by salad juice exposure.

**FIG 2:**
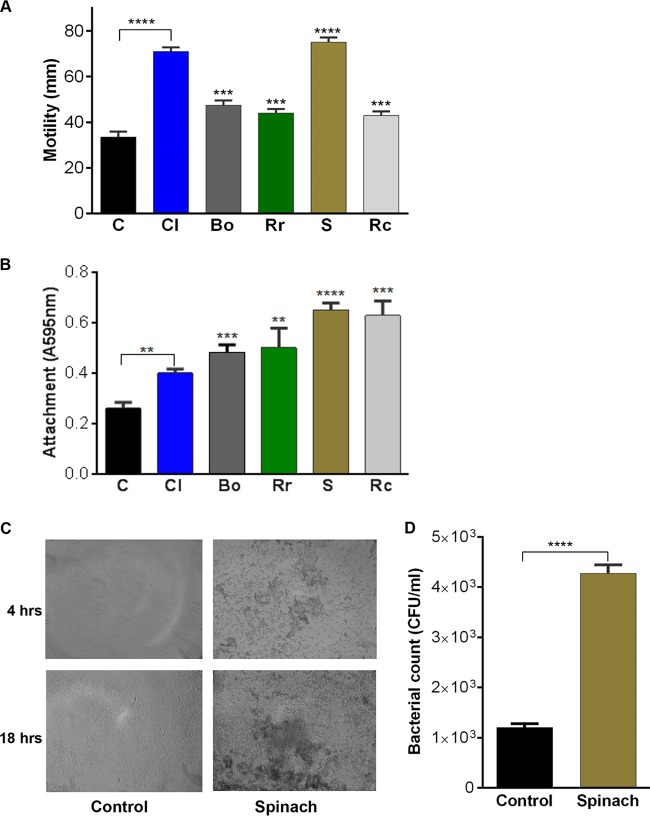
Salad extracts increase Salmonella motility and biofilm formation. (A) Salmonella motility at room temperature in 2% (vol/vol) salad leaf juice-supplemented DMEM relative to unsupplemented controls (*P* ≤ 0.01). Key: C, control (no additions); Cl, cos lettuce; Bo, baby green oak lettuce; Rr, red romaine lettuce; S, spinach; Rc, red chard; *n* = 5. (B) Initial *in vitro* attachment of Salmonella in water in the presence of salad juice relative to unsupplemented controls (*P* ≤ 0.001). Initial inocula were 10^6^ CFU/ml; values shown are means of 4 data points; *n* = 3. Key: C, control (no additions); Cl, cos lettuce; Bo, baby green oak lettuce; Rr, red romaine lettuce; S, spinach; Rc, red chard. The various leaf juices were supplemented as 2% (vol/vol) additions; *n* = 3. (C) Light microscopy image showing biofilm formation of Salmonella treated for 4 and 18 h with incubation at room temperature with 2% spinach juice–DMEM; *n* = 6. (D) Histogram showing that 2% spinach extract in water enhances Salmonella attachment to spinach leaves after a 30-min incubation at room temperature; values shown are means of 6 data points; *n* = 3. **, *P* ≤ 0.01; ***, *P* ≤ 0.001; ****, *P* ≤ 0.0001.

Motility and biofilm formation have both been shown to be important in the colonization and persistence of Salmonella on fresh produce (29, 30). Motility is known to contribute to leaf internalization by promoting entry into plant wounds and stomata (3, 29–31). We examined the motility of Salmonella in the presence of the salad leaf juices and show in Fig. 2A that a 2% addition of any of the juices significantly increased motility relative to unsupplemented control results, with spinach juice being the most potent additive (*P* ≤ 0.001).This finding agrees with those reported by Kroupitski et al., who showed that Salmonella bacteria exhibit a specific tropism toward the cut surfaces of salad leaves, where plant juices are present in the greatest abundance (29).

Motility and attachment to surfaces are important for Salmonella pathogenesis and for the ability of the bacteria to associate with fresh produce (29, 30, 32–34). Therefore, the effect of salad juice on attachment to a polystyrene plate surface was analyzed, as salads are often contained within plastic bags (3) (Fig. 2B). It can be seen that, relative to controls, all the salad juices increased the attachment of Salmonella (*P* ≤ 0.001). We used water as our incubation medium for the attachment assays to more closely represent the typical salad bag environment; however, attachment was enhanced in all of the liquid media tested, including the media which contained host-like serum (data not shown). We also looked at the effects of juice on the later stages of biofilm formation. The images in Fig. 2C show the typical appearance of spinach juice-treated Salmonella after 4 and 18 h of room temperature incubation and that juice exposure markedly enhances exopolysaccharide production and cell-cell attachment, both of which are important in biofilm recruitment (3). Similar results were seen with all the salad juices tested in the experiments whose results are shown in Fig. 2A (data not shown). In terms of attachment to salad leaves, Fig. 2D shows that even a 30-min exposure to a salad juice significantly enhanced pathogen attachment to salad leaves with an affinity that was resistant to several water washings (*P* ≤ 0.0001). These results may explain why enteric pathogens tend to accumulate on plant sites where there is fluid leakage from wounds (3), as a study using E. coli found that the attachment to cut salad leaf surfaces was stronger than the attachment to intact ones (34).

### Salad juice enhances colonization of salad leaves by Salmonella.

**FIG 3:**
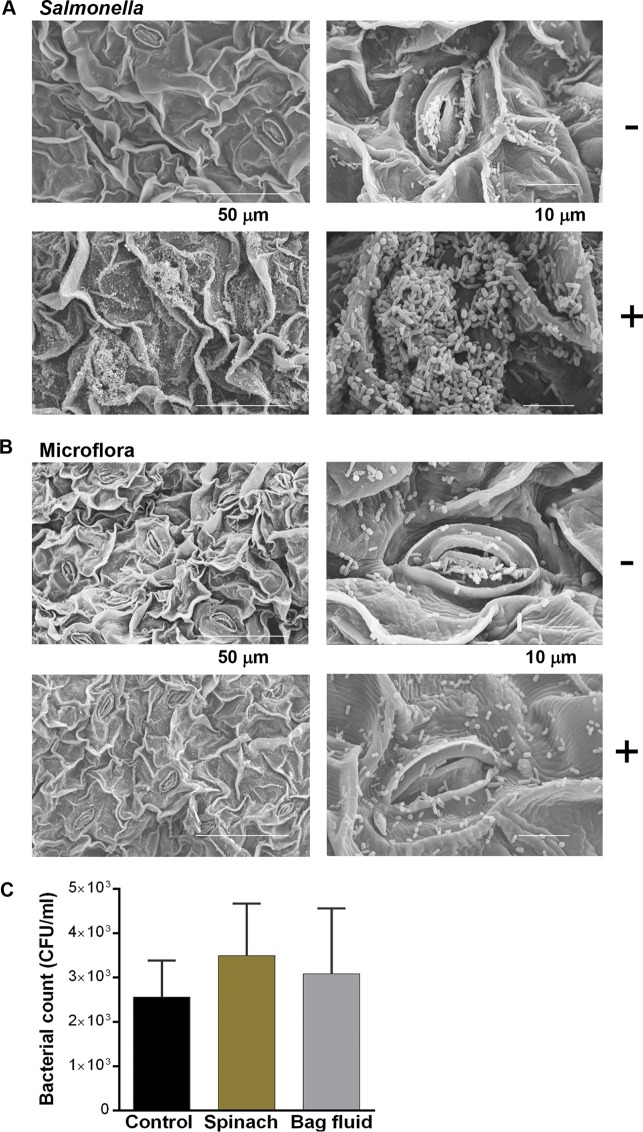
Spinach juice increases Salmonella colonization of salad leaves. (A) Representative scanning electron micrographs of untreated (−) and 2% (vol/vol) spinach juice-exposed (+) Salmonella seeded at 10^5^ CFU/ml onto 5-by-5-mm sections of spinach leaves as described in Materials and Methods. (B) Attachment profiles of the leaf microbiota in the absence (−) and presence (+) of 2% (vol/vol) spinach juice; *n* = 3. Scale bars are shown in white at the bottom of each set of images. (C) *In vitro* growth responses of the salad leaf microbiota (inoculated at 10^2^ CFU/ml) to spinach juice (2%) and spinach bag fluid (20%); *P* > 0.05; *n* = 8.

We also investigated whether salad fluid exposure directly influenced the capacity of Salmonella to colonize salad leaves. Scanning electron micrographs (SEM) of spinach leaves incubated in water with or without 2% juice inoculated with 10^5^ CFU/ml Salmonella for 24 h are shown in Fig. 3A (this higher inoculum was used to ensure that the growth rates and final growth levels of controls and salad juice-supplemented cultures were similar). It can be seen that, in comparison with nontreated controls, the association of Salmonella with the plant surface was greatly increased in the presence of the salad juice (*P* ≤ 0.001). Increasing magnifications of images of the spinach leaves showed that the large increase in the numbers of bacteria attached when the spinach juice was present was particularly associated with the stomata, which appeared to be a preferred colonization point; this is a finding that has also been seen in a number of other studies (8, 29, 30).

Salad leaves have diverse microbial populations which are largely derived from their culture environment (35). Therefore, to more realistically reproduce salad leaf storage conditions, we did not exclude the endogenous microbes from the investigations whose results are shown in Fig. 3. Use of SEM to profile the response of the endogenous microbiota of the spinach leaves to juice exposure (Fig. 3B) showed there was much less stimulation of growth or formation of biofilms by other members of the microbiota by the salad juice than of the pathogen. Figure 1 and 2 show clearly that Salmonella was highly responsive to salad juice in terms of enhancement of growth. In contrast, when we analyzed the growth responsiveness of the microbiota, there were only minimal increases in cell numbers over the control level (*P* > 0.05) (Fig. 3C). This suggests that the endogenous salad leaf microbes are naturally accustomed to fluids released from the leaves and so have adapted to their presence, which, in terms of salad leaf colonization, would be an advantage for a more juice-responsive pathogen such as Salmonella.

### Salad bags may be a colonization site for enteric pathogens.

**FIG 4:**
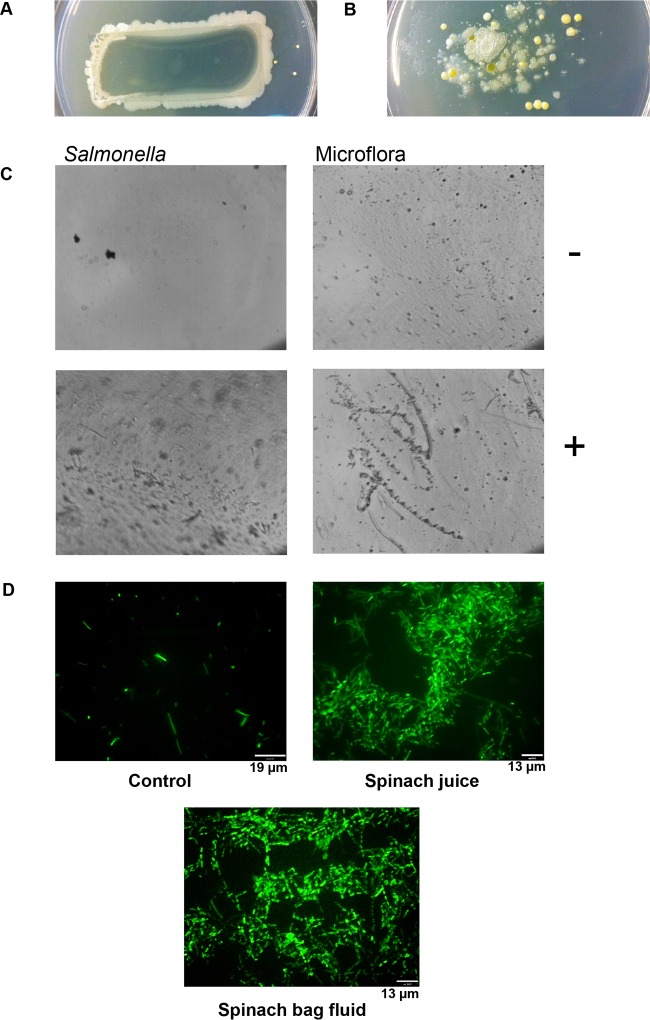
Salad juice increases colonization of salad bags by Salmonella. (A and B) Typical pictures of a salad bag microbiota. Panel A shows microbes growing from the salad bag and panel B the diversity of microbes transferred from a bag imprint onto Luria agar. (C) Light microscopy images of Salmonella (seeded at 10^5^ CFU/ml) and endogenous microbiota colonization of salad bag plastic in water in the absence (−) and presence (+) of 2% spinach juice; images were captured using a light microscope (bright field) set at ×60 magnification; *n* = 8. (D) Blue light microscopy images (×60 magnification) of 10^5^ CFU/ml GFP-Salmonella incubated in water in the absence (Control) and presence of 2% spinach juice or 20% spinach bag fluid; *n* = 3.

A bag composed of a synthetic plastic (such as polyethylene terephthalate or polypropylene) is the usual container for ready-to-eat salad leaves (3). When we cultured sections of a commercial salad bag in liquid culture media or took Luria agar plate imprints from bag sections, we discovered that the inner surface of the bag possessed a diverse attached microbiota population (illustrated in Fig. 4A and B), which we found to be present on the interior of all the salad leaf bags that we purchased. We therefore examined the salad container for its potential to be a platform for biofilm formation by pathogenic bacteria by incubating bag sections in water with and without salad juice. Figure 4C presents a light microscopy image showing that Salmonella attachment to the salad bag was low unless a salad juice was present (in this case, 2% spinach juice). In contrast, in the absence of additional factors, the endogenous microbiota colonized the bag more effectively than the pathogen; however, the exposure to salad juice did not increase microbiota biofilm formation to the same extent as for the Salmonella. We also noted that addition of the Salmonella inoculum seemed to displace many of the microbiota from the bag surface (Fig. 4A), even though the Salmonella themselves attached poorly unless a salad juice was present. This finding is in marked contrast to the work of Cooley et al., who showed that the presence of the microbiota could reduce pathogen colonization of lettuce (36). We were not able to analyze the architecture of the salad bag biofilms by scanning electron microscopy (SEM) as the composition of the bag polymer was unstable in the presence of the solvents used to dehydrate samples. However, Fig. 4D shows a set of green fluorescent protein (GFP)-Salmonella microscopy images recorded under blue light and reveals the extensive nature and some of the architecture of the biofilm produced in the presence of salad leaf juice. The results shown in Fig. 4D illustrate that leaf juice traces within the salad bag fluids were similarly able to induce the growth of a substantial wash-resistant biofilm on the plastic bag surface. This suggests that the salad bag container could be an important bacterial attachment site, even though the salad container has not been factored into considerations of what influences pathogen colonization or retention (3).

### Salad bag fluids enhance Salmonella growth in host-like media.

**FIG 5:**
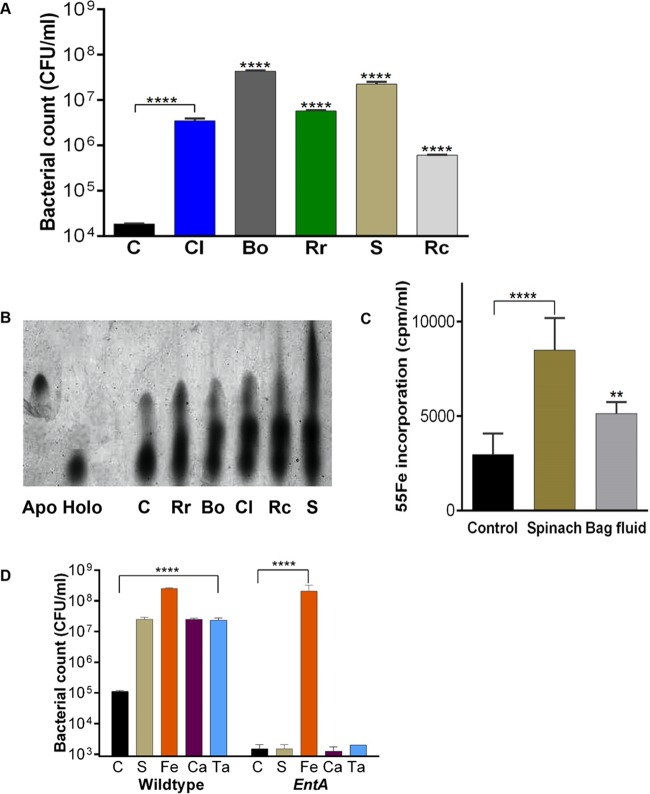
Salad bag fluids enhance Salmonella growth in host-like media. (A) Histograms showing the salad juice response growth responses of Salmonella in serum-SAPI medium. Bacteria were inoculated at 10^2^ CFU/ml and incubated for 18 h at 37°C. Values shown are means of 6 data points; relative to controls, all data values have a *P* value of ≤0.001; *n* = 3. Key: C, control (no additions); Cl, cos lettuce; Bo, baby green oak lettuce; Rr, red romaine lettuce; S, spinach; Rc, red chard. (B) Urea gel showing that salad extracts (2% vol/vol) can remove iron from holo-transferrin (Holo); *n* = 5. Key: C, control (no additions); Cl, cos lettuce; Bo, baby green oak lettuce; Rr, red romaine lettuce; S, spinach; Rc, red chard. (C) Histograms showing the uptake of transferrin-complexed ^55^Fe by Salmonella in the absence and presence of 2% spinach juice (Juice) (*P* ≤ 0.0001) and 20% spinach bag fluid (Bag fluid) (*P* ≤ 0.001; *n* = 3). (D) Histograms comparing the serum-SAPI growth response profile at 37°C of a Salmonella enterobactin mutant with that of the wild type in the presence of salad juice and salad leaf dietary catechols. Values shown are means from 3 data points; *n* = 3. Key: C, control (no additions); S, 2% (vol/vol) spinach juice; Fe, 100 μM ferric nitrate; Ta, 100 μM tannic acid; Ca, 100 μM chlorogenic acid. **, *P* ≤ 0.01; ****, *P* ≤ 0.0001.

The data in Fig. 1 to 4 show that exposure of Salmonella to salad leaf juices appears to enhance its capacity to grow, colonize, and persist on salad leaves. We were also curious as to what the exposure of Salmonella to salad juice might mean in the infection context if the pathogen and the salad leaves were coconsumed. We therefore examined whether salad juice increased the capacity of Salmonella to grow in host-like serum-containing media (37). The results presented in Fig. 5A show that a 2% addition of the salad juice was stimulatory to Salmonella growth in serum-SAPI (6.25 mM NH_4_NO_3_, 1.84 mM KH_2_PO_4_, 3.35 mM KCl, 1.01 mM MgSO_4_; 2.77 mM, containing 30% [vol/vol] adult bovine serum), inducing an up to 3-log increase in bacterial numbers over control levels (*P* ≤ 0.001). Higher juice concentrations such as 5% were slightly more stimulatory, and lower doses less so, though up to a 1 log improvement was observed at a salad juice level of 0.2%. In terms of the mechanism of this stimulation, serum is bacteriostatic as a consequence of Fe restriction caused by the presence of transferrin, a high-affinity ferric iron-sequestering glycoprotein that plays a protective role in blood and other secretory fluids in mammals (38), and so we looked at iron provision as a possible explanation. This idea was supported by reports that catechols such as chlorogenic and tannic acids are abundant in salad leaves and have a siderophore-like structure (39–41). Their function is to aid plant survival by limiting free iron availability to colonizing plant pathogens and by protecting plant biological molecules from oxidative damage caused by Fe-generated free radicals. The urea gel presented in Fig. 5B shows that compounds within the salad juices were able to remove iron from transferrin at doses equivalent to those which stimulated growth in serum-SAPI (Fig. 5A). We therefore examined Salmonella uptake of transferrin iron (from ^55^Fe-labeled transferrin) in the presence of salad juice. Figure 5C shows that Salmonella was able to assimilate significantly more of the transferrin iron (in the form of ^55^Fe) when the salad juice was present (*P* ≤ 0.0001). We also found that leaf juice traces within the bag fluids were similarly able to supply Fe from transferrin (Fig. 5C) (*P* ≤ 0.01). Because this data set suggested the involvement of altered iron availability, we repeated our serum-SAPI salad juice growth assays with a Salmonella *entA* mutant which is defective in enterobactin synthesis and therefore in Fe uptake. Figure 5D shows that, in marked contrast to the wild-type strain, the siderophore mutant was unresponsive to all of the juices tested (an example using 2% spinach juice is shown). To determine if salad catechols were the stimulatory factors in salad juices, we compared the responses of wild-type Salmonella and the *entA* mutant to chlorogenic and tannic acids. Figure 5D shows that, unlike wild-type Salmonella, the *entA* mutant was completely unresponsive to any of the catechols tested (*P* ≤ 0.0001).

Siderophore synthesis has been shown to be important for fresh produce colonization, as Hao et al. (42) found that a Salmonella enterobactin synthesis mutant (*entB*) was unable to colonize lettuce leaves. Hao et al. (42) also demonstrated that fluids released from alfalfa seedlings directly induced S. enterica expression of enterobactin and salmochelin. Our finding that mutation of *entA* abolishes salad juice responsiveness suggests that the ability to respond to leaf juice is an essential part of salad leaf colonization. Salmonella siderophore production is also important for growth within Fe-limited animal host fluid as it enables bacterial access to the iron within proteins such as transferrin and lactoferrin (3).

In conclusion, in this report we have shown that even very dilute levels of salad juices are potent stimulators of Salmonella growth and salad leaf attachment and colonization and that the presence of the existing leaf microbiota does not prevent pathogen attachment. Salad juice exposure also has the potential to enhance Salmonella virulence which may contribute to the severity of the food infections obtained from salad leaves (3). A very recent study has shown that salad leaf juices also enhance the growth and biofilm formation of pathogenic E. coli (43). These findings, combined with our study results, emphasize the importance of preventing fresh produce contamination, and so attention has to be paid to maintaining strict hygiene standards at all levels of salad leaf production.

## MATERIALS AND METHODS

### Bacterial strains.

The strains which were used in this project were the S. enterica SL1344 wild-type strain and a mutant of that strain that is defective in the synthesis of enterobactin (Δ*entA*) (source, R. Haigh). GFP-labeled S. enterica SL1344 (GFP-Salmonella) was used in some of the biofilm assays and was made according to the method of Ma et al. (44). Strains were routinely maintained on Luria agar plates supplemented with 50 μg/ml kanamycin in the case of the GFP strain and stored at 4°C for up to 2 weeks, after which strain stocks were repropagated from −80°C culture stocks.

### Culture media and growth analyses.

Salmonella cultures for inoculation were grown in 5 ml of Luria broth and for analysis of growth responsiveness to salad extracts in triplicate 1-ml volumes of sterile water. For analyses in host-like media, we used serum-SAPI (6.25 mM NH_4_NO_3_, 1.84 mM KH_2_PO_4_, 3.35 mM KCl, 1.01 mM MgSO_4_; 2.77 mM, containing 30% [vol/vol] adult bovine serum) as it is more reflective of the challenging conditions within a host (38, 45). Salmonella was inoculated into water or serum-SAPI to give a cell count of approximately 10^2^ CFU/ml. Leaf juice growth response assays in serum-SAPI (supplemented with the doses described in the text) were incubated statically for 18 h at 37°C in a humidified 5% (vol/vol) CO_2_ incubator. After incubation, cultures were mixed and aliquots withdrawn for estimation of growth by plate count analysis (45). To model the behavior of Salmonella within the salad bag environment, the bacteria were also cultured in sterile water supplemented with 2% (vol/vol) salad juices at room temperature or refrigeration (4°C) temperature as described for the individual experiments. For the analysis of growth responses of the salad leaf microbiota to salad juices, 5-mm-by-5-mm sections of leaf were subjected to vortex mixing for 30 s in 1 ml of sterile water to release surface-attached bacteria. The products of these washings (100 μl) were then plated onto Luria agar plates, and organism numbers were counted after 24 h incubation at room temperature (which we found to be the preferred growth temperature of the salad microbiota). This information was retrospectively used to calculate the inoculum size (approximately 10^2^ CFU/ml). This strategy was necessary because of variability in the microbiota levels and because some species grew faster than others. The microbiota inocula were added to quadruplicate 1-ml volumes of fresh sterile water supplemented with salad juice or salad bag juice and incubated at room temperature for 24 h as described in the text. For all other growth assays, plate counts were carried out in at least triplicate, and all experiments were performed on at least two separate occasions.

### Salad juice preparation.

Salad leaves (cos lettuce, baby green oak lettuce, red romaine lettuce, spinach, and red chard) were sorted into types from commercially prepared bagged mixes and 10 g was extracted by grinding performed for 90 s with a pestle and mortar, followed by centrifugation for 30 min at 5,000 × *g* to remove large particulate debris. The extract (now termed “juice”) was further centrifuged for 10 min at 13,000 × *g* and was sterilized by filtration through a 0.2-μm-pore-size syringe filter. We typically obtained the following average volumes of juice per 10 g of salad leaf: for cos lettuce, 8 ml; for baby green oak lettuce, 3.5 ml; for red romaine lettuce, 6 ml; for spinach, 4 m; and for red chard, 6 ml. Tests to determine growth and motility effects showed that the activity within the sterile salad leaf juices was stable for several weeks at 4°C, but for longer term storage, to maintain stability of activity, juices were kept at −80°C. For collection of fluids from within salad bags, bags were tilted at a 90° angle (as shown in Fig. 1B), pegged to a stand, and stored at 4°C overnight. Fluids (typically 1 to 2 ml/bag) were collected from the corner of the bag and were filter sterilized as described for salad leaf juices. To test their growth stimulatory effects, the juices were added at a dose range of 5 to 20% (vol/vol) to 10^1^ CFU/ml Salmonella inoculated into sterile water; the cultures were incubated statically at 4°C for up to 5 days and growth levels enumerated using plate count analysis.

### Motility and biofilm analysis.

Twitching motility assays were performed in Dulbecco's modified Eagle's medium (DMEM) (46) solidified with 0.3% agar with or without 2% salad juice. Salmonella (5 μl of a 10^8^ CFU/ml overnight culture) was stab inoculated into the agar and incubated at room temperature for 24 h, and the migration distance was measured.

Analysis of the effect of salad juice exposure on Salmonella biofilm formation was also performed. To quantify initial attachment, bacteria were cultured in triplicate at room temperature in 96-well plates in water or bacterial culture media (DMEM or Luria broth) as described for the individual experiments. After incubation, the nonadherent cells were removed and the adhered cells washed gently twice with phosphate-buffered saline (PBS), followed by staining with 0.2% crystal violet. After 15 min at room temperature, the stain was removed, the cultures were washed 3 times with water, and the adherent stain eluted with 20:80 (vol/vol) acetone-ethanol. Cell/biofilm biomass was quantified by measurement of the levels of eluted crystal violet stain at 595 nm using a microplate reader (Bio-Rad). Correction for background staining was made by subtracting the value for the crystal violet bound to the uninoculated medium controls.

Enumeration of the effects of salad juice exposure on Salmonella-salad leaf association was performed by measuring attachment to the salad leaves. An inoculum of 10^5^ CFU/ml Salmonella was added to sterile water containing 2% (vol/vol) salad leaf juice and three 5-mm-by-5-mm spinach leaf pieces; the control consisted of water only. The inoculated spinach pieces were incubated statically at room temperature for 30 min, after which the leaves were removed aseptically, washed once by dipping into 5 ml of sterile water, and then transferred to another 5 ml of sterile water and subjected to vortex mixing for 90 s. To account for endogenous microbiota, the enumeration of attached Salmonella was performed by plating the wash liquids on selective Rapid Salmonella agar plates (Bio-Rad). For SEM visualization of Salmonella colonization of spinach leaves, leaf sections were similarly prepared with an inoculum of 10^5^ CFU/ml. Following inoculation, the leaves were incubated at room temperature for 24 h before washing and fixing in 2.5% glutaraldehyde–10 mM Tris-HCl (pH 7.0) was performed. After fixation, leaf sections were washed and dehydrated in a graded ethanol series to 100%, followed by infiltration with hexamethyldisilazane before air drying. The dry leaf sections were mounted onto aluminum stubs, sputter coated with gold under cool conditions, and imaged by SEM using a Hitachi S3000H scanning electron microscope (37).

For analysis of Salmonella biofilm formation on salad bag plastic (composed of either polyethylene terephthalate or polypropylene—the two plastic types functioned similarly in our assays), the bag was surface sterilized with ethanol and then cut into approximately 20-mm-by-5-mm sections using sterile scissors. These were then placed into sterile water containing no additions or 2% spinach juice. The GFP-labeled Salmonella was then inoculated into the water at 10^5^ CFU/ml, and the culture was incubated at room temperature for 24 h. To account for the endogenous microbiota that might have colonized the plastic surface, identical assays of bag sections alone were performed. After incubation, plastic sections were washed twice in sterile water and visualized for biofilm by light microscopy, as we found that the SEM solvent treatments dissolved the plastic bag sections.

### Transferrin analysis.

In order to determine whether the salad juices were stimulating growth in serum-SAPI medium by removing iron from serum transferrin, a transferrin iron chelation assay was carried out. Diferric (holo-) transferrin (50 μg in 100 mM Tris-HCl [pH 7.5]) was incubated for 24 h at 37°C with 2% (vol/vol) salad juice or water only. Transferrin samples were analyzed for iron removal by electrophoresis in a 6 M urea polyacrylamide gel for 4 h at a constant voltage of 70 V, followed by staining for 10 min in 0.1% Coomassie brilliant blue–10% glacial acetic acid–40% methanol. Gels were destained in 7.5% methanol–5% acetic acid (38). For investigation of whether salad juices could directly increase Salmonella uptake of transferrin iron, sterile SAPI medium buffered with 100 mM Tris-HCl (pH 7.5) and containing ^55^Fe-labeled transferrin (1.5 × 10^5^ cpm/ml), prepared as described by Freestone et al. (38), was supplemented with 2% sterile salad leaf juice or 20% of spinach salad bag fluid or an equivalent volume of water. Washed Salmonella was added at 1 × 10^8^ CFU/ml and incubated at 37°C in a 5% CO_2_ incubator for 18 h, after which bacteria were harvested by centrifugation at 10,000 rpm for 10 min and then washed in PBS and assayed for cell number determinations by plate counting and for ^55^Fe incorporation using scintillation counting as described previously (38).

### Data analysis.

Where appropriate, statistical analysis was carried out using an unpaired *t* test for comparison of 2 groups and a 2-way analysis of variance (ANOVA) with the Bonferroni test for multiple comparisons using Graph Pad Prism Program version 6. Statistical significance was indicated by a *P* value of less than 0.05 (in terms of statements of significance: *, *P* ≤ 0.05; **, *P* ≤ 0.01; ***, *P* ≤ 0.001; and ****, *P* ≤ 0.0001).
